# The influence of gender, media, and internet usage on adolescents' fast food perception and fluid intake

**DOI:** 10.1186/s41043-023-00426-x

**Published:** 2023-08-02

**Authors:** Ahmad R. Al-Haifi, Nayef Y. Bumaryoum, Balqees A. Al-Awadhi, Fahad A. Alammar, Rasha H. Ashkanani, Hazzaa M. Al-Hazzaa

**Affiliations:** 1 Department of Food and Nutrition Science, College of Health Sciences, PAAET, Showaikh, Kuwait; 2 Department of Home Economics, Basic Education College, PAAET, Showaikh, Kuwait; 3 Lifestyle and Health Research Center, Health Sciences Research Center, Princess Nourah Bint Abdulrahman University, Riyadh, Saudi Arabia

**Keywords:** Adolescent, Kuwait, Media impact, Obesity, Weight loss, Gender difference, Fast foods, Fluid consumption

## Abstract

**Background:**

Understanding the factors that influence adolescent’s perception of fast food and their fluid consumption is crucial for designing effective nutrition education programs tailored to this population. This study aimed to evaluate the associations of sex and the use of media and the internet with adolescents' perception of fast foods and the amount of fluid consumption.

**Methods:**

A cross-sectional survey was conducted on school adolescents between the ages of 15 and 18 years in Kuwait, using a multistage stratified random sampling method (N = 706 adolescents; 343 boys and 363 girls). A pre-tested and specifically designed self-report questionnaire covering several measures such as: (a) types of foods that are considered fast foods; and (b) participant’s fluid consumption. Body weight and height were measured using calibrated medical scales. Body mass index (BMI) was computed. The adolescents were stratified by sex into two groups: boys and girls, for the purpose of analysis.

**Results:**

Seven out of 14 food choices showed significant sex differences (p values ranged from 0.016 to < 0.001) in the adolescents’ responses to whether they perceived such food choice as fast food or not. Although differences were found between sexes, the majority of the listed fast foods were correctly recognized as fast foods by the adolescents. In addition, there were significant differences between males and females in the amount of daily drinks (ml/week) consumed from full fat milk (males = l197.1 ± 27,652.1 and females = 1662.8 ± 2221, p = 0.013), sugar-sweetened beverage (males = 2350.8 ± 3324.3 and females = 3088.9 ± 3701.1, p = 0.004), and energy drinks (males = 429.5 ± 1117.2 and females = 267.6 ± 733.8, p = 0.037). Compared to adolescents who seldom or do not watch TV or use the internet, those who engage in these sedentary activities are less likely to classify pizza (aOR (95% CI) = 0.660 (0.440–0.990), p = 0.045), grilled meat (aOR (95% CI) = 0.674 (0.477–0.954), p = 0.026), fried egg sandwiches (aOR (95% CI) = 0.617 (0.425–0.894–0.189), p = 0.011), and rice (aOR (95% CI) = 0.598(0.409–0.875), p = 0.008) as fast foods.

**Conclusion:**

The influence of TV and internet use on adolescent’s ability to accurately identify fast foods has been observed. Findings indicate the need for increased fast food nutrition education programs that are tailored towards adolescents. The study recommends further research to enhance consumer awareness of foods and drinks among adolescents in the State of Kuwait.

## Background

Social media offers various advantages for personal users and organizations to communicate, socialize, and market their products. When used appropriately, social media is an effective tool to communicate and to share food safety news and best practices [1]. Adolescents who typically adopt new dietary habits that may persist into their adult years [2], may adversely affect their health and nutritional status [3, 4]. Studies indicate that a higher frequency of fast-food (mostly high in saturated fats, added sugar, and sodium) consumption is positively associated with less healthy food choices and a higher body mass index (BMI) compared to lower frequency of fast-food consumption [5–7]. However, most of the previous studies have focused on the frequency of fast food consumption rather than on its definition. Fast food has been defined by Bender and Bender [8] as a “general term used for a limited menu of foods that lend themselves to production-line techniques; suppliers tend to specialize in products such as hamburgers, pizzas, chicken, or sandwiches”. Fast food has become an important part of the diet/cuisine in Arab Gulf countries which is believed to be fueled by advancements in the region’s economy and increase in western franchise branding [5, 9–11].

It has been found that the perception of food as fast food or non-fast food is significantly different between sexes [12, 13]. In Kuwait, fast food can be classified as Western, Eastern, or Local food. For instance, Burgers and Noodles may be categorized as Western and Eastern fast foods, respectively, while local fast foods include sandwiches (e.g., shawarma) and Indian fast food. Musaiger indicated that Kuwaiti foods were more likely to be considered fast food by adults if eaten as a sandwich [12]. Increased fast food consumption has been linked to a diet that is high in calories, saturated fat, sugar, and sodium, as well as to body fatness, weight gain, and increased body mass index [14], which could negatively impact health. These nutritional components, coupled with a sedentary lifestyle, may contribute to the development of various health problems.

It has been suggested that soft drinks could be a significant factor in the observed increase in obesity rates [14, 15]. Studies have shown a positive association between soft drink consumption and energy intake among children and adolescents [16]. This may be due to overconsumption, which is more likely to occur when energy is consumed in liquid form, and because soft drinks often represent additional energy rather than a replacement alternative [17]. Added sugar, such as that found in soft drinks or energy drinks, provides the diet with only empty calories. This means that it supplies energy without any other essential nutrients.

Consequently, lack of nutrition knowledge among adolescents and definitions of fast foods are complex integrations of attitudes, beliefs, health consciousness, body weight status, sex, and environmentally led social behaviors [12, 18, 19]. Unhealthy food advertising plays a significant role in the popularity of such food. Regular consumption of fast food products can also lead to obesity, diabetes, heart disease, and various types of skin cancers [19, 20]. To avoid consuming Unhealthy food and maintain a healthy diet, it is important to develop self-awareness and resist the temptation for unhealthy foods. Undoubtedly, unhealthy foods are often high in fat, salt, sugar, and calories, and provide little to no nutritional value. Common fast foods include salted snack foods, fried fast food, and carbonated drinks. Fast foods have become a major problem and many countries are taking action-banning such unhealthy food advertising in children's programmers, removing it from schools and even imposing a fat tax [20]. In recent studies, media exposure, particularly on social media platforms, has been found to significantly influence the dietary choices of children and adolescents [21]. Therefore, the current study was aimed to investigate the effects of sex, the use of media and the internet on adolescent’s perception of fast foods and fluid intake consumption.

## Methods

### Design and sampling

A cross-sectional survey was conducted on school adolescents aged 15 to 18 years in the state of Kuwait over a period of five months during the 2019 academic year. The students were selected using a multistage stratified cluster random sampling method, as previously described [22]. The stratification was based on sex (boys vs. girls’ schools), private versus public schools, and geographical/administrative locations (governorates) in the State of Kuwait. A predetermined sample size of 368 participants for each sex group was calculated, so that the population proportion was assumed to be at 0.60 with a margin of error of 5% and a confidence level of 95%. Collected data included a total of 706 adolescents, comprising 343 boys and 363 girls. Response rate was very high reaching 99% of the selected students. The sample for this study was obtained from students enrolled in both public and private schools across Kuwait. Initially, schools were randomly selected and stratified according to sex, type of school (private and public), and location. A total of 14 schools were selected, comprising 12 public schools and 2 private schools, with 7 schools for boys and 7 schools for girls. Subsequently, one classroom from each grade (i.e., 10th, 11th, and 12th) was randomly selected in each school, resulting in a total of 42 classrooms surveyed. The study protocol was approved by the Ministry of Education in Kuwait before data collection commenced. Informed consents were obtained from all participating students and consents were attained from their parents if they were younger than 18 year-olds.

### Survey description

A pre-tested, specifically designed, and validated self-reported questionnaire [22, 23] was used to assess the exposure to mass media (television (TV) and Internet use) as well as fast food perception and fluid Intake. Examples of the questions (with response option of “yes” or “no”) include: How often do you read the newspapers, watch television, or use the Internet per day? What food choices are considered fast food or not? Does reading the newspapers or watching television influence your food or drink choice? Other questions that were included in the questionnaire, but not reported in the present study, encompass the impact of media on body weight-loss, the perception of body thinness, and parental or friend's perception of body weight [22].

### Anthropometric measurements

Body weight was measured to the nearest 100 g and height to the nearest 0.1 cm using calibrated medical scales (Seca 875 Weight Scale & Seca 213 Stadiometer) by trained faculty staff and their students. All measurements were conducted with minimal clothing and without shoes. Body mass index (BMI) was computed as the ratio of weight in kilograms divided by the squared height in meters. The extended International Obesity Task Force (IOTF) age- and sex-specific BMI cutoff reference standards were used to classify underweight, normal weight, and overweight or obesity relative to the adolescent’s age [24]. To facilitate analysis, the adolescents were classified by sex as boys and girls.

### Statistical analysis

Data were entered into an SPSS data file, checked, cleaned, and analyzed using IBM-SPSS program, version 22 (Chicago, IL, USA). Sex, age, BMI, media and internet use are independent variables, while food and drink choices represent dependent variables. Descriptive statistics were obtained for all variables and reported as means and standard deviations or percentages. Differences between males and females in anthropometric measurements were tested using t-test for independent samples. Chi-Square tests of proportions were used to test differences in selected variables related to the impact of media on adolescents’ decision to lose weight or their perception of body thinness relative to sex. Multivariate analyses (MANCOVA), while controlling for age, were used to test differences in the total amount of drinks consumed, relative to sex. In addition, logistic regression analysis, adjusted for age, was used to test differences in the perception of selected food choices (fast food or not) against sex or media use among Kuwaiti adolescents. The adjusted odds ratio (aOR) and confidence intervals were reported. Alpha level was set at 0.05 and *p*-value less than alpha level was considered significant.

## Results

Table 1 presents the distribution of fast food perceptions among male and female adolescents. The table highlights the proportion of participants who considered burgers, fried potatoes, pizza, fried chicken, shawarma, hot dogs, and falafel as fast foods, while revealing that a significant number of respondents did not classify grilled meat, pie, fried egg sandwiches, chickpeas, Koskosi, and rice as fast foods. Furthermore, sex differences were observed in the perception of specific food choices. Significant sex differences were noted for fried potato (p = 0.001), grilled meat (p = 0.002), Shawarma (p = 0.009), Fataer (p = 0.001), hot dog (p = 0.016), chickpeas (p = 0.021), and Koskosi (p = 0.001).

**Table 1:** Adolescents’ responses to which of the following food choices is fast food or not?

Kind of foods	Males	Females	p value*
*All kinds of burgers*			
Yes	92.7	95.3	0.149
No	7.3	4.7	
*Fried potato*			
Yes	82.5	90.6	**0.001**
No	17.5	9.4	
*Pizza*			
Yes	79.3	76.6	0.385
No	20.7	23.4	
*Fried chicken*			
Yes	80.8	84.6	0.180
No	9.2	15.4	
*Grilled meat*			
Yes	42.6	31.4	**0.002**
No	57.4	68.6	
*Shawarma (grilled meat or chicken sandwich)*			
Yes	70.6	61.2	**0.009**
No	29.4	38.8	
*Fataer (cheese or thyme pie)*			
Yes	55.0	43.0	**0.001**
No	45.0	57.0	
*Foul (fava beans)*			
Yes	49.3	43.8	0.145
No	50.7	56.2	
*Falafel sandwich (made of fava beans and chickpeas)*			
Yes	59.2	52.1	0.057
No	40.8	47.9	
*Hot dog*			
Yes	72.9	64.5	**0.016**
No	27.1	35.5	
*Fried eggs sandwich*			
Yes	49.6	43.8	0.125
No	50.4	56.2	
*Chickpeas (Hummus)*			
Yes	46.1	37.5	**0.021**
No	53.9	62.5	
*Koskosi (Moroccan food)*			
Yes	48.7	36.1	**0.001**
No	51.3	63.9	
*Rice*			
Yes	34.7	28.7	0.084
No	65.3	71.3	

Bold values indicate statistically significant *p*-values

*Chi Squares tests for the differences in proportion

Results of multivariate analysis, while controlling for the effect of age, for the total amount of drinks (ml/week) consumed by the adolescent relative to sex are shown in Table 2. There were significant differences between males and females in the number of daily drinks consumed from full fat milk (p = 0.005), sugar-sweetened beverage (p = 0.005), and energy drinks (p = 0.024). The mean (SD) daily fluid intake was 2818.5 (2000.9) ml. Furthermore, water appeared as the most popular beverage among both male and female adolescents. Notably, as compared to their male counterparts, a higher proportion of female participants reported higher water consumption in accordance with their age and weight. Fruit juices and fruit drinks were the second most popular liquids after water. Sweetened beverages and full-fat milk were in second and third place, respectively, in terms of consumption frequency. Conversely, energy drinks and protein shakes had the lowest consumption rates among adolescent consumers.

**Table 2:** Multivariate analysis of drinks consumption (ml/week) by sex, controlling for age and body weight in adolescents

Variable	Males	Females	p value*
Water	2700.1 ± 2047.1	2803.3 ± 1660.3	Age: **0.005**; weight: 0.610; Gender: 0.351
Fruit juice	2778.8 ± 2798.7	2479.1 ± 2475.1	Age: 0.077; weight: 0.115; Gender: 0.399
Fruit drinks	2875.2 ± 2932.4	3188.5 ± 2764.1	Age: 0.187; weight: 0.550; Gender: 0.109
Full fat milk	2197.1 ± 2765.1	1662.8 ± 2221.6	Age: 0.273; weight: 0.655; Gender: **0.013**
Low fat milk	1553.3 ± 2380.9	1334.4 ± 2133.3	Age: 0.451; weight: 0.593; Gender: 0.314
Skimmed milk	1043.5 ± 1899.9	1046.1 ± 1930.2	Age: 0.132; weight: 0.548; Gender: 0.880
Sugar-sweetened beverage	2350.8 ± 3324.3	3088.9 ± 3701.1	Age: 0.933; weight: 0.437; Gender: **0.004**
Diet beverage	692.5 ± 1086.7	659.5 ± 1090.8	Age: 0.361; weight: 0.800; Gender: 0.787
Tea with milk	1424.0 ± 2289.6	1329.2 ± 2142.6	Age: 0.091; weight: 0.559; Gender: 0.768
Coffee with milk	787.4 ± 1304.6	961.3 ± 1276.6	Age: 0.670; weight: 0.999; Gender: 0.092
Tea or coffee	1032.4 ± 2037.2	1229.8 ± 2405.8	Age: 0.375; weight: 0.564; Gender: 0.189
Herbal tea	890.0 ± 1952.8	704.1 ± 1664.7	Age: 0.562; weight: 0.058; Gender: 0.533
Beer drinks	774.8 ± 1773.7	653.5 ± 1664.7	Age: 0.934; weight: 0.506; Gender: 0.513
Energy drinks	429.5 ± 1117.2	267.6 ± 733.8	Age: 0.427; weight: 0.914; Gender: **0.037**
Protein shake	251.5 ± 639.7	328.6 ± 1306.9	Age: 0.204; weight: 0.109; Gender: 0.725

Bold values indicate statistically significant *p*-values

*Wilks’ Lambda p values for the main effects for each of age = 0.565, weight = 0.803, and gender = 0.002

Table 3 presents the outcomes of the logistic regression analysis, which was adjusted for age, examining the perception of selected food choices as fast food or not fast food among Kuwaiti adolescents, stratified by sex. Male adolescents were less inclined to classify fried potato as fast food compared to their female counterparts (aOR = 0.489; 95% CI 0.299–0.800; p = 0.004), and more likely to call the following food choices as fast foods: grilled meat (aOR = 1.431; 95% CI 1.014–2.021; p = 0.042), hot dog (aOR = 1.502; 95% CI 1.035–2.180; p = 0.032), and Koskosi (Moroccan food) (aOR = 1.565; 95% CI 1.117–2.193; p = 0.009).

**Table 3:** Logistic regression analysis of food choice (fast or not) vs. sex among Kuwaiti adolescents, adjusted for age

Variable	Males versus Females *			
	aOR	(95% CI)	SEE	***p-****value*
Age	1.073	0.909—1.266	.085	0.406
All kinds of burgers (no = ref)	1.00			
Yes	0.973	0.474—2.000	0.367	0.942
Fried potato (no = ref)	1.00			
Yes	0.489	0.299—0.800	0.251	**0.004**
Pizza (no = ref)	1.00			
Yes	1.174	0.790—1.745	0.202	0.427
Fried chicken (no = ref)	1.00			
Yes	0.829	0.532—1.292	0.226	0.408
Grilled meat (no = ref)	1.00			
Yes	1.431	1.014—2.021	0.176	**0.042**
Shawarma (grilled meat or chicken sandwich) (no = ref)	1.00			
Yes	1.060	0.733—1.533	0.188	0.758
Fataer (cheese or thyme pie) (no = ref)	1.00			
Yes	1.410	0.990—2.008	0.181	0.057
Foul (fava beans) (no = ref)	1.00			
Yes	0.856	0.563—1.301	0.214	0.467
Falafel sandwich (made of fava beans and chickpeas) (no = ref)	1.00			
Yes	0.989	0.648—1.509	0.216	0.959
Hot dog (no = ref)	1.00			
Yes	1.502	1.035—2.180	0.190	**0.032**
Fried eggs sandwich (no = ref)	1.00			
Yes	1.087	0.753—1.570	0.187	0.656
Chickpeas (Hummus) (no = ref)	1.00			
Yes	1.031	0.718—1.480	0.184	0.869
Koskosi (Moroccan food) (no = ref)	1.00			
Yes	1.565	1.117—2.193	0.172	**0.009**
Rice (no = ref)	1.00			
Yes	1.155	0.791—1.686	0.193	0.457
Chinese foods (no = ref)	1.00			
Yes	1.067	0.722—1.578	0.200	0.745
Indian foods (no = ref)	1.00			
Yes	1.130	0.752—1.697	0.207	0.556
Mexican foods (no = ref)	1.00			
Yes	0.808	0.500—1.304	0.244	0.382
Italian foods (no = ref)	1.00			
Yes	0.772	0.498—1.196	0.223	0.247

Bold values indicate statistically significant *p*-values

*Female was used as a reference category

*aOR* adjusted odds ratio; *CI* confidence interval; *ref* reference category; *SEE* standard error

Table 4 presents the results if logistic regression analysis, adjusted for age and sex, for selected food choices as fast food or not among Kuwaiti adolescents relative to TV use or internet use. Compared to those who seldom or do not watched TV or used the internet, those adolescents who watch TV or use the internets were less likely to classify pizza (aOR = 0.660; 95% CI 0.440–0.990; p = 0.045), grilled meat (aOR = 0.674; 95% CI 0.477–0.954; p = 0.026), fried eggs sandwich (aOR = 0.617; 95% CI 0.425–0.894; p = 0.011), and rice (aOR = 0.598; 95% CI 0.409–0.875; p = 0.008) as fast foods.

**Table 4:** Logistic regression analysis of food choice (fast or not) versus TV/internet use among Kuwaiti teens

Variable	TV use versus seldom/none*			
	aOR	(95% CI)	SEE	p value
Age	0.810	0.685–0.975	.085	**0.013**
Gender (female = ref)	1.00			
Male	1.165	0.849–1.599	0.161	0.344
Pizza (no = ref)	1.00			
Yes	0.660	0.440–0.990	0.207	**0.045**

Variable	Internet use versus seldom/none*			
	aOR	(95% CI)	SEE	p value
Age	0.912	0.771–1.078	.086	0.281
Gender (female = ref)	1.00			
Male	1.521	1.107–2.090	0.162	**0.010**
Grilled meat (no = ref)	1.00			
Yes	0.674	0.477–0.954	0.177	**0.026**
Fried eggs sandwich (no = ref)	1.00			
Yes	0.617	0.425–0.894	0.189	**0.011**
Rice (no = ref)	1.00			
Yes	0.598	0.409–0.875	0.194	**0.008**

Bold values indicate statistically significant *p*-values

*Seldom/none use was used as a reference category for TV use or internet use

*aOR* adjusted odds ratio; *CI* confidence interval; *ref* reference category; *SEE* standard error

## Discussion

The study findings indicate that there were sex differences among adolescents in the perception of seven out of 18 selected food choices as whether they are fast foods or not? Results have also shown that although differences were found between sexes, both girls and boys were not aware of which foods are considered fast food or not. There were significant differences between males and females in the amount of daily drinks consumed from full fat milk, sugar-sweetened beverage, and energy drinks. The results of this study are consistent with earlier research showing a significant relationship between sex and fast food choices and knowledge [25, 26].

Fast food consumption is particularly high in males and often accompanied by other unhealthy lifestyle behaviors including a high level of tobacco and media consumption with a concurrent deficiency in fruit and vegetable consumption [26]. Interpersonal factors associated with higher sweetened beverage intake include low socio-economic status [26].

The results of the current study agree with the findings of Hansstien et al. [27], which aimed to compare the relationship between new media exposure and fast food consumption among Chinese children and adolescents in schools, specifically in Chinese rural and urban areas. [27]. The findings indicated a significant relationship between new media exposure and fast food consumption in both rural and urban areas in China. Both behaviors are potentially associated with negative mental and physical health outcomes [27]. Therefore, we suggest that further research and implementation of surveillance systems are needed to monitor the consequences and promote health promotion policies.

On the other hand, mass media, including social media use, may be one of the main reasons that impact adolescents understanding of fast food and what is considered unhealthy [27]. It has been suggested previously by Kent et al., that around 50% of food marketing that is targeted towards adolescents on social media is directed towards the consumption of fast-food and sweetened beverages which may in turn have an impact on their perceptions of which foods are considered fast-food [28]. According to recent research by Fleming-Melici et al., involving 1564 teenagers in the US, increased exposure to social media and television is associated with unhealthy food choices among adolescents [29]. These findings underscore the need for targeted fast food nutrition education programs for this population.

While the current study benefitted from a sufficient sample size and the use of a validated questionnaire, there were some limitations that should be noted. For instance, the lack of socio-economic data limits us to control for such variables. The presence of socioeconomic data on the participants could have provided important insights into the underlying factors impacting perceptions of media use on food and drink choices. Also, self-reported behavioral data may introduce bias, with the potential for under- or overestimation. Furthermore, perceptions within this specific age group, namely adolescents, may differ from those of other age cohorts, as teenagers tend to display a heightened sensitivity during this developmental stage, particularly in relation to body image perception.

## Conclusion

Our study highlights the significant gender-based differences in the perception of fast food and beverage consumption among Kuwaiti adolescents. Additionally, our findings reveal a high percentage of adolescents consuming sugar-sweetened drinks, indicating a need for targeted interventions to promote healthy beverage choices. The present study highlights the necessity for further research to enhance consumer awareness of food and beverage choices among Kuwaiti adolescents. It is through these future investigations, that a deeper understanding of the factors influencing fast food preferences and the effectiveness of intervention strategies can be revealed. This knowledge will play a vital role in developing a comprehensive and tailored approach to promote healthier eating habits among adolescents in Kuwait.

## Data Availability

All data generated or analyzed during this study are included in this published article. Any additional data will be available from the corresponding author upon reasonable request.

## Appendix

A copy of the questionnaire in Arabic language.
